# Pterostilbene Alleviates Chlorpyrifos-Induced Damage During Porcine Oocyte Maturation

**DOI:** 10.3389/fcell.2021.803181

**Published:** 2021-12-21

**Authors:** Lili Guo, Yongda Zhao, Yanjun Huan

**Affiliations:** ^1^ College of Veterinary Medicine, Qingdao Agricultural University, Qingdao, China; ^2^ National Risk Assessment Laboratory for Antimicrobial Resistance of Microorganisms in Animals, College of Veterinary Medicine, South China Agricultural University, Guangzhou, China

**Keywords:** chlorpyrifos, pterostilbene, nuclear maturation, cytoplasmic maturation, oocyte

## Abstract

Chlorpyrifos (CPF), a widely used organophosphate pesticide, is reported to severely impair mammalian reproductive system. Pterostilbene (PTS), an effective free radical scavenger, is considered as beneficial for mammalian reproduction. However, the toxicity of CPF on oocyte maturation and whether PTS can eliminate the detrimental effect of CPF on oocytes remain unclear. Here, porcine oocytes were applied to investigate the potential effect and possible mechanism of CPF and PTS during oocyte maturation. This work demonstrated that CPF significantly delayed the meiotic progression and decreased the polar body extrusion by disturbing spindle assembly and chromosome alignment and causing DNA damage in oocytes (*p* < 0.05). And, CPF significantly impaired oocyte cytoplasmic maturation by inducing the high level of reactive oxygen species and decreasing glutathione content (*p* < 0.05). Moreover, CPF significantly triggered embryo apoptosis and reduced the blastocyst rate and cell number following parthenogenetic activation (*p* < 0.05). Whereas CPF-exposed oocytes were treated with PTS, these defects caused by CPF were obviously rescued, and oocyte maturation and subsequent embryonic development were also significantly ameliorated (*p* < 0.05). In conclusion, these results revealed that CPF exerted the toxic effect on porcine oocytes, while PTS effectively alleviated CPF-induced damage on oocytes. This work provides a potential strategy to protect oocyte maturation in mammalian species.

## Introduction

Reproductive toxicity is considered as a most common pathological cause of mammalian infertility (Mourikes and Flaws, 2021). It has been reported that with the wide use of pesticides in the control of agriculture and residential pests, organophosphate (OP) insecticides, a largest and most diverse group, cause the adverse effects on the reproductive system of consumers (Fucic et al., 2021). An increasing number of reports has demonstrated that OP insecticides destroy the functions of ovary and testis, and increase the risk of infertility (Nishi and Hundal, 2013; Abdel-Razik et al., 2021; Ubaid Ur Rahman et al., 2021).

CPF, a conventional broad-spectrum chlorinated OP insecticide, is widely used to control pests to enhance agricultural production, however, continuous CPF exposure is detrimental to mammalian health due to the cumulatively toxic effect (Ubaid Ur Rahman et al., 2021). Numerous studies have revealed that CPF disturbs mammalian physiological and biochemical functions and further impairs the reproductive system (Nishi and Hundal, 2013; Chebab et al., 2017; Sai et al., 2020). Previous studies have also suggested that the toxicity induced by CPF could be associated with the induction of reactive oxygen species (ROS) and the broken of antioxidant-prooxidant balance, and the over production of free radicals due to CPF exposure has been shown to cause the oxidation of thiol groups of glutathione (GSH) which is defined as the intracellular redox buffer and protects cells against oxidative stress, increase DNA damage, disturb cell cycle, and result in cell apoptosis or death (Li et al., 2015; Chen et al., 2018; Salyha and Salyha, 2018). In addition, CPF exposure alters the contents of antioxidant enzymes such as glutathione peroxidase, superoxide dismutase and catalase (Barski et al., 2011; Akpa et al., 2021). Moreover, accumulation of CPF has also been reported in follicles (Al-Hussaini et al., 2018). These available reports suggest that CPF exposure could disrupt female reproduction.

Oocyte maturation, referring to nuclear and cytoplasmic maturation, is an essential event in mammalian reproduction, and determines the progress of reproductive technologies such as *in vitro* fertilization (IVF), somatic cell nuclear transfer (SCNT) and intracytoplasmic sperm injection (ICSI) (Huan et al., 2015b). During nuclear maturation, the precise regulation of spindle assembly and chromosome alignment is required to ensure the normal meiosis, and cytoplasmic maturation is the spatial distribution of organelles, and also includes the function of the redox system (Lee et al., 2016; Jochems et al., 2021). When oocytes are exposed to environmental pollutants, spindle assembly and chromosome alignment are disturbed, and the balance of redox system is destroyed (Chen et al., 2020; Shi et al., 2021). OP insecticides have been reported to destroy the antioxidant system to produce ROS and reduce GSH, and further lead to DNA damage in oocytes, and CPF is also shown to affect oocyte maturation (Nandi et al., 2011; Li et al., 2015; Jiang et al., 2021). However, the possible mechanism underlying the effect of CPF on mammalian oocyte maturation remains unclear.

Antioxidants are usually considered as an effective strategy to alleviate the oxidative damage induced by environmental pollutions including CPF (Chebab et al., 2017; Salyha and Salyha, 2018; Fereidouni et al., 2019). PTS, a natural bioactive compound primarily derived from pterocarpus marsupium heartwood, blueberries and grapes, possesses various biological functions including antioxidation, anti-inflammatory and anticancer (Ullah et al., 2019). Growing evidences have demonstrated that PTS is a strong scavenger of free radicals and safeguards cells against DNA damage (Acharya and Ghaskadbi, 2013; Zhang H. et al., 2020). PTS has also been reported as a potent Nrf2 (Nuclear factor erythroid2-related factor 2) activator and possess the antioxidant property through the Nrf2-mediated pathway (Bhakkiyalakshmi et al., 2016; Ullah et al., 2019). In addition, studies have demonstrated that PTS can reduce cell damage caused by free radicals to enhance embryo development, and is suggested to improve oocyte maturation (Ullah et al., 2019; Sosa et al., 2020). Moreover, PTS has been shown to alleviate the toxic effect of environmental pollutions including pesticides (Zhou J. et al., 2019; Tang et al., 2021). Therefore, it is reasonable to assume that PTS could take a protective role against the defects induced by CPF during oocyte maturation.

Pigs own the great research value in agriculture, biomedicine and basic research, and also share many similar physiological characteristics with humans (Yang et al., 2021), thus porcine oocytes can be an ideal model to investigate the potential effect of environmental compounds on mammalian reproduction. Although several studies have reported the toxicity of CPF and the protective effect of PTS on mammalian reproductive system (Fereidouni et al., 2019; Ullah et al., 2019; Jiang et al., 2021), it remains unclear that how CPF exposure affects porcine oocyte maturation and whether PTS can protect oocytes against CPF. Here, to investigate the potential effect and possible mechanism of CPF and PTS during oocyte maturation, porcine oocytes were applied, and the results demonstrated that CPF exerted the toxic effect on porcine oocytes, while PTS effectively alleviated CPF-induced damage on oocytes. This work provides a potential strategy to protect oocyte maturation in mammalian species.

## Materials and Methods

Chemicals were purchased from Sigma Aldrich Corporation, and plasticware was obtained from Nunclon, unless otherwise stated.

### Oocyte Maturation and Treatment

Oocyte maturation has been described in our previous study (Huan et al., 2015b). Briefly, porcine ovaries were collected from a local slaughterhouse and transported to laboratory. Follicles were aspirated, and follicular contents were washed with HEPES-buffered Tyrode’s lactate. Cumulus-oocyte complexes (COCs) were recovered, washed and cultured in maturation medium for 42 h. Then, COCs were vortexed in hyaluronidase to remove cumulus cells, and oocytes with the visible polar body, regular morphology and homogenous cytoplasm were counted to calculate oocyte maturation rate and applied in the subsequent experiments.

For oocyte treatment, COCs were cultured in maturation medium with CPF at the concentration of 0.00, 0.05, 0.10, 0.25, 0.50 or 1.00 μM, PTS at the concentration of 0.00, 0.10, 0.25, 0.50, 0.75 or 1.00 μM, or both CPF and PTS at the optimal concentration for 42 h, respectively. The group of oocytes untreated was named as the CON group, the group of oocytes treated with CPF at the optimal concentration was the CPF group, the group of oocytes treated with PTS at the optimal concentration was the PTS group, and the group of oocytes treated with both CPF and PTS at the optimal concentration, respectively, was the CPT group.

### Parthenogenetic Activation and Embryo Development

The procedure for parthenogenetic embryo development has been described in our previous report (Huan et al., 2015b). Briefly, matured oocytes were placed between electrodes, and activated by the direct pulse in fusion medium. Then, embryos were washed and cultured in porcine zygote medium-3 (PZM-3) for the subsequent development.

The cleavage and blastocyst rates were observed and evaluated at 48 and 156 h, respectively, and blastocysts were collected and applied in the subsequent experiments.

### Oocyte Nuclear Maturation Progression and Blastocyst Cell Number

Oocyte nuclear maturation progression and blastocyst cell number were analyzed by nuclear staining (Wang et al., 2021). Oocytes and blastocysts were fixed with paraformaldehyde, and stained with Hoechst 33,342. After staining, oocytes and blastocysts were washed and mounted on slides. Then, oocyte nuclear status and blastocyst cell number were examined under a fluorescence microscope.

### Spindle Morphology, Chromosome Alignment and DNA Damage

The detection of spindle morphology, chromosome alignment and DNA damage has been described previously (Miao et al., 2021). Briefly, matured oocytes were treated with acidic Tyrode’s solution to remove zona pellucida, fixed with 4% paraformaldehyde, permeabilized with 1% Triton X-100, and blocked with 1% BSA. Then, oocytes were incubated with anti-β-tubulin or anti-γH2AX (Abcam) antibody, and labeled with FITC conjugated secondary antibody. Then, oocytes were stained with PI to detect chromosome alignment or Hoechst 33,342. After staining, oocytes were washed, mounted on slides and examined to analyze the spindle or chromosome morphology and the fluorescence intensity for DNA damage in each oocyte using Image-Pro Plus 6.0.

### ROS Level

ROS detection has been reported with a ROS Assay Kit (Beyotime) (Jiang et al., 2021). Briefly, matured oocytes were incubated with 10 μM 2′,7′-dichlorofluorescin diacetate (DCFH-DA) at 38.5°C for 30 min. Then, oocytes were washed and examined to measure the fluorescence intensity in each oocyte.

### GSH Content

The measurement of GSH content has been reported in our previous study (Huan et al., 2015b). Briefly, for GSH staining, matured oocytes were incubated with 5 μM 4-chloromethyl-6,8-difluoro-7-hydroxycoumarin (CMF2HC, Invitrogen) for 30 min and manipulation medium for another 30 min. Then, oocytes were washed and examined to analyze the fluorescence intensity in each oocyte. For quantitative measurement, GSH content in matured oocytes was determined using a GSH assay kit (Beyotime). After oocytes were frozen and thawed three times using liquid nitrogen and 37°C water, GSH content was measured by the 5,5′-dithiobis (2-nitrobenzoic acid) oxidized glutathione reductase recycling assay. According to the standard curve, total GSH amounts in samples were calculated and divided by the number of oocytes to get GSH content per oocyte.

### Gene Expression

Measurement of gene expression with quantitative real-time PCR has been applied in our previous study (Huan et al., 2015a). Briefly, total RNA was extracted from matured oocytes or blastocysts using a RNeasy Micro Kit (Qiagen). Reverse transcription was performed using a PrimeScript^®^ RT Reagent Kit (TaKaRa). For quantitative real-time PCR, reactions were performed in the 96-well optical reaction plate using a SYBR^®^ Premix ExTaq™ II kit (TaKaRa). For every sample, the CT values were obtained from three replicates. The primers were presented in Supplementary Table S1. Relative gene expression levels were analyzed using the 2^−ΔΔCT^ method.

### Blastocyst Apoptosis

Detection of blastocyst apoptosis using the TUNEL method with an *In Situ* Cell Death Detection Kit (Roche) has been described in our previous work (Wang et al., 2021). Briefly, blastocysts were fixed with paraformaldehyde, permeabilized with Triton X-100, incubated in the terminal deoxynucleotidyl transferase mediated dUTP nick end labeling reaction medium, and stained with Hoechst 33,342. Then, the number of apoptotic cells per blastocyst was counted, and the apoptotic rate per blastocyst was analyzed.

### Statistical Analysis

Differences in data (mean ± SEM) were analyzed with the SPSS statistical software (Wang et al., 2021). Statistical analyses of data concerning oocyte maturation, embryo development, blastocyst cell number, oocyte nuclear status, spindle assembly, chromosome alignment, DNA damage, ROS, GSH, embryo apoptosis, and gene expression were performed with *t*-test when the comparison was made between two groups or one-way analysis of variance when there were more than two groups. For all analyses, differences were considered statistically significant when *p* < 0.05.

## Results

### Effects of CPF and PTS on Oocyte Nuclear Maturation

**FIGURE 1:**
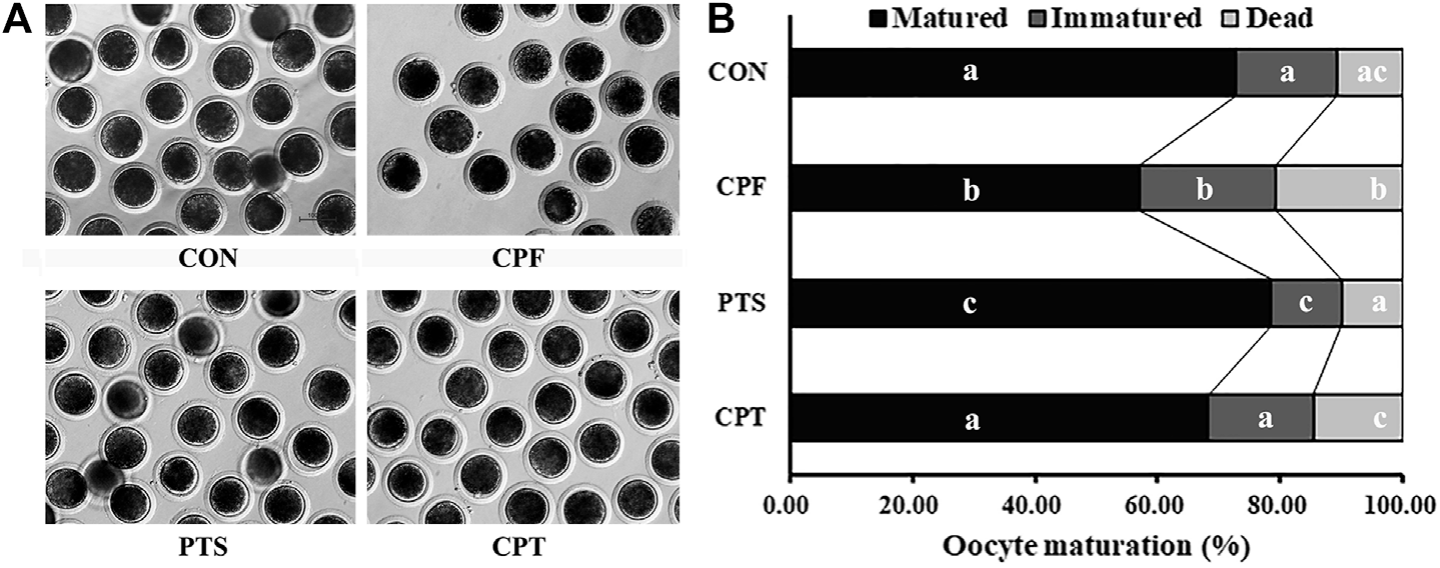
Effect of CPF and PTS on oocyte maturation. **(A)**, oocyte morphology (×40). **(B)**, oocyte maturation rate. ^a-c^Values for a given state with different superscripts differ significantly (*p* < 0.05).

To clarify the effect of CPF and PTS on oocyte nuclear maturation, the polar body extrusion (PBE), as the marker of oocyte nuclear maturation, was examined. The results displayed that when oocytes were treated with the various concentration CPF, 0.25, 0.50 or 1.00 μM CPF significantly decreased the rate of oocyte maturation, and increased the percentage of oocyte death compared with the CON group (Supplementary Table S2, *p* < 0.05). Considering that 0.50 or 1.00 μM CPF belonged to a higher dose for oocytes, and resulted in the even more lower maturation rate, 0.25 μM CPF (the CPF group) was chosen for the subsequent treatment. For the dose dependent effect of PTS on oocyte maturation, the results showed that 0.50 or 0.75 μM PTS significantly upregulated the maturation rate compared with the CON group, and 0.50 μM PTS was better (Supplementary Table S3, *p* < 0.05), thus, 0.50 μM PTS (the PTS group) was applied in the further experiment. In the CPT group, oocyte maturation rate was significantly upregulated and the percentages of immatured and dead oocytes were significantly downregulated compared with the CPF group, and no significant maturation differences of oocyte maturation rate were observed compared with the CON group (Figure 1 and Supplementary Table S4, *p* < 0.05). Thus, CPF exposure blocked oocyte PBE, while PTS was benefit for oocyte nuclear maturation, and protected oocyte PBE against CPF.

### Effects of CPF and PTS on Oocyte Nuclear Maturation Progression

**FIGURE 2:**
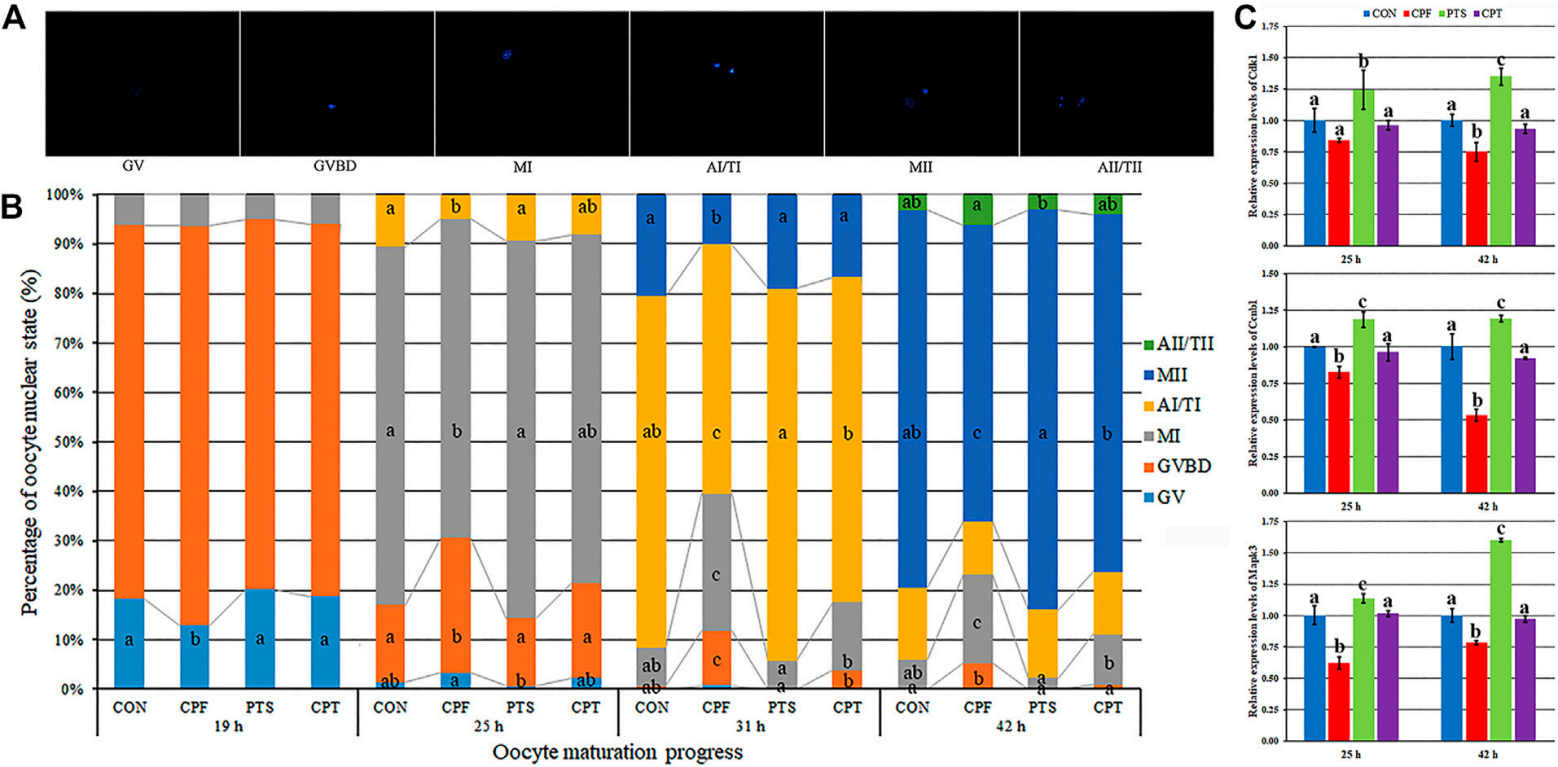
Effect of CPF and PTS on oocyte nuclear maturation progression. **(A)**, nuclear morphology (×400). **(B)**, percentage of nuclear state. **(C)**, relative expression of MPF and MAPK related genes. ^a-c^Values for a given state or group with different superscripts differ significantly (*p* < 0.05).

PBE is associated with oocyte nuclear maturation progression. During *in vitro* maturation, oocytes carried out meiosis, meaning that germinal vesicle (GV), germinal vesicle breakdown (GVBD), the first metaphase (MI), the first anaphase/telophase (AI/TI) and the second metaphase (MII) successively appeared, and the second anaphase/telophase (AII/TII) by spontaneous activation occasionally occurred (Figure 2A). For the time point of meiotic event, oocytes entered GVBD at 19 h, MI at 25 h, AI/TI at 31 h and MII at 42 h (Supplementary Table S5). When compared with the CON group, CPF resulted in the significantly lower rates of MI at 25 h, AI/TI at 31 h, and MII at 42 h, and higher rates of GVBD at 25, 31 and 42 h and MI at 31 and 42 h (*p* < 0.05), while the percentages of AI/TI at 31 h and MII at 42 h were significantly upregulated and the percentages of GVBD at 25, 31 and 42 h, and MI at 31 and 42 h were obviously downregulated in the CPT group compared with the CPF group (*p* < 0.05), and no significant differences of oocyte nuclear state were observed between the CPT group and the CON group (Figure 2B and Supplementary Table S6). When the transcription levels of cell cycle genes related to maturation promoting factor (MPF) and mitogen-activated protein kinase (MAPK) were detected at 25 and 42 h, compared with the CON group, the CPF group displayed the significantly downregulated expression levels of Ccnb1 and Mapk3 at 25 h and Cdk1, Ccnb1, and Mapk3 at 42 h (*p* < 0.05), while the CPT group showed no significant differences of Cdk1, Ccnb1, and Mapk3 expression, and the significantly upregulated transcripts of Ccnb1 and Mapk3 at 25 h and Cdk1, Ccnb1, and Mapk3 at 42 h were observed in CPT group compared with the CPF group (Figure 2C, *p* < 0.05). These results suggested that CPF disrupted oocyte nuclear maturation progression, while PTS restored this progression of CPF-exposed oocytes.

### Effects of CPF and PTS on Oocyte Spindle Assembly, Chromosome Alignment and DNA Damage

**FIGURE 3:**
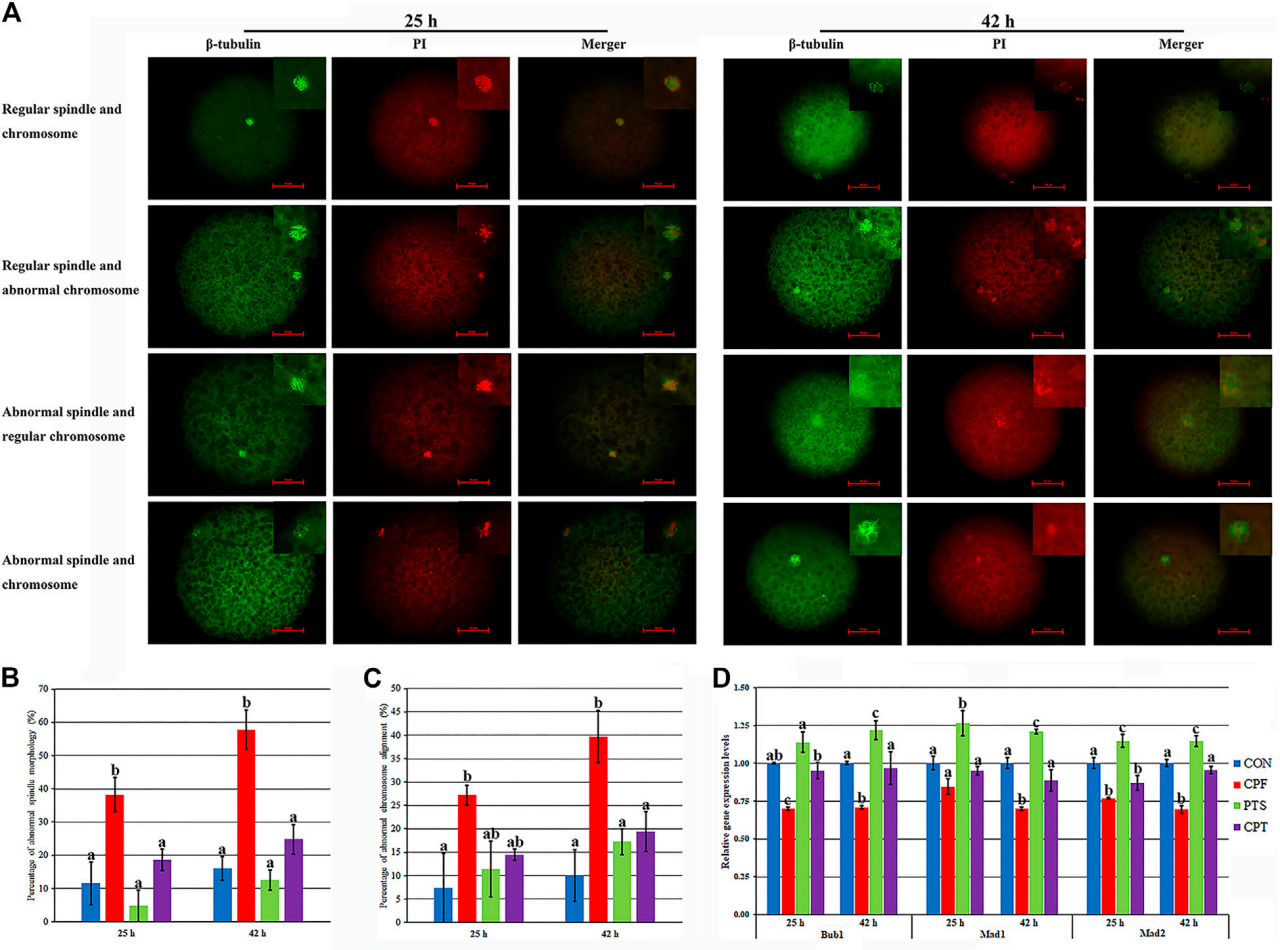
Effect of CPF and PTS on oocyte spindle assembly and chromosome alignment. **(A)**, spindle or chromosome morphology (×400). **(B)**, percentage of abnormal spindle morphology. **(C)**, percentage of abnormal chromosome alignment. **(D)**, relative expression of SAC related genes. The number of oocytes for spindle and chromosome detection in the CON, CPF, PTS or CPT group was 23, 30, 19 or 21 at 25 h, or 45, 34, 47 or 39 at 42 h, respectively. ^a-c^Values for a certain group with different superscripts differ significantly (*p* < 0.05).

Spindle assembly and chromosome alignment are key factors determining PBE. Here, oocyte spindle and chromosome morphologies were detected after CPF or/and PTS treatment. Compared with the CON group, CPF induced a significantly higher percentage of abnormal spindle and chromosome morphologies (*p* < 0.05), while the CPT group showed the similar percentages of abnormal spindle and chromosome morphologies, and the percentages of abnormal spindle morphology at 25 h and aberrant spindle and chromosome morphologies at 42 h were significantly downregulated in the CPT group compared with the CPF group (Figures 3A–C, *p* < 0.05). When the mRNA levels of spindle assembly checkpoint (SAC) related genes (Bub1, Mad1, and Mad2) were detected (Figure 3D), the significantly downregulated levels of Bub1 and Mad2 at 25 h and Bub1, Mad1, and Mad2 at 42 h in the CPF group but only Mad2 at 25 h in the CPT group were observed compared with those in the CON group, and the CPT group displayed the significantly upregulated transcription levels of Bub1 at 25 h, and Bub1, Mad1, and Mad2 at 42 h than the CPF group (Figure 3D, *p* < 0.05). Thus, CPF disrupted oocyte spindle assembly and chromosome alignment, while PTS protected oocytes against these defects induced by CPF.

**FIGURE 4:**
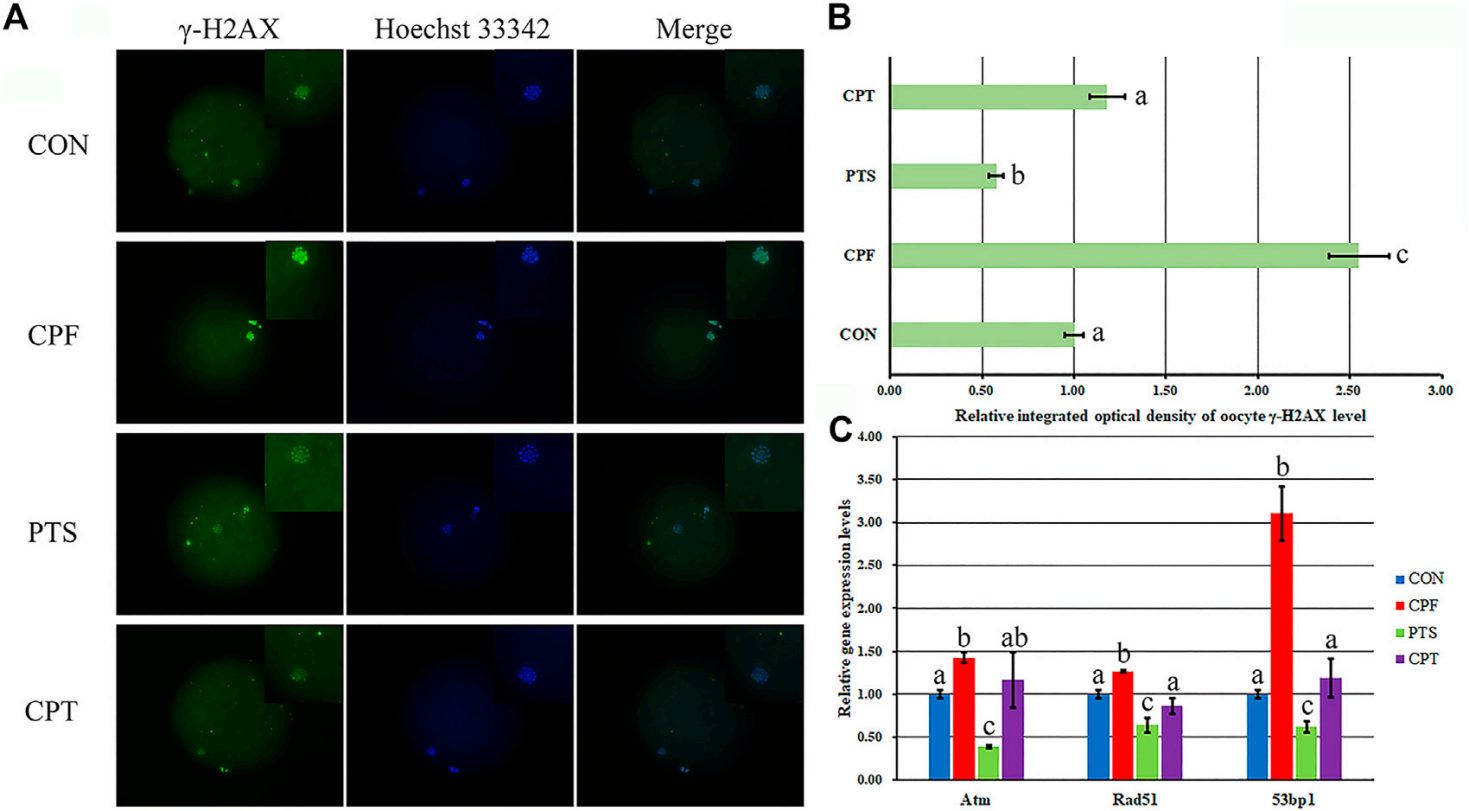
Effect of CPF and PTS on oocyte DNA damage. **(A)**, fluorescence of γ-H2AX (×400). **(B)**, relative integrated optical density of γ-H2AX. **(C)**, relative expression of DNA repair related genes. The number of oocytes for DNA damage detection in the CON, CPF, PTS or CPT group was 20, 19, 20 or 22, respectively. ^a-c^Values for a certain group with different superscripts differ significantly (*p* < 0.05).

DNA damage inhibits oocyte maturation, and DNA double strand break (DSB) reflects the degree of DNA damage. Here, γ-H2AX in response to DNA DSB was examined to assess DNA damage (Figure 4). Compared with the CON group, the CPF group displayed a significant increase of γ-H2AX level (*p* < 0.05), while the CPT group exhibited the similar γ-H2AX level, and a significant reduction of γ-H2AX level was observed in the CPT group compared with the CPF group (*p* < 0.05). Moreover, compared with the CON group, the transcription levels of DNA repair related genes including Atm, Rad51, and 53bp1 significantly increased in the CPF group (*p* < 0.05), but were similar in the CPT group, and compared with the CPF group, the CPT group displayed the significantly lower expression of Rad51 and 53bp1 (*p* < 0.05). These results suggested that CPF caused DNA damage to oocytes, while PTS relieved the defect.

### Effects of CPF and PTS on the Contents of Oocyte ROS and GSH

**FIGURE 5:**
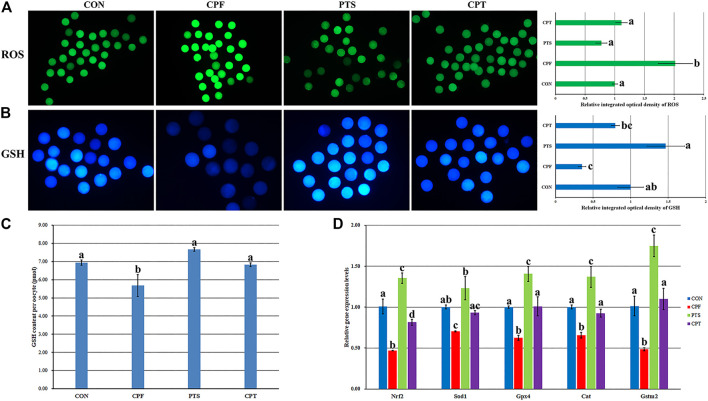
Effect of CPF and PTS on oocyte ROS and GSH content. **(A)**, fluorescence and relative integrated optical density of ROS (×40). **(B)**, fluorescence and relative integrated optical density of GSH (×100). **(C)**, GSH content per oocyte. **(D)**, relative expression of redox related genes. ^a-d^Values for a certain group with different superscripts differ significantly (*p* < 0.05).

The balance between ROS and GSH is essential to regulate oocyte cytoplasmic maturation. ROS induces oxidative stress and causes damage to oocytes, while GSH, which functions as an antioxidant to defend against the damage of oxidative stress, is required for oocyte competence. Here, the contents of ROS and GSH and the expression levels of redox genes including Nrf2, Sod1, Gpx4, Cat, and Gstm2 were investigated (Figure 5). Compared with the CON group, the CPF group displayed a significant increase of ROS level and decrease of GSH content (*p* < 0.05), while the CPT group took on the similar levels of ROS and GSH (Figures 5A–C), and the CPT group showed a significant decrease of ROS level and increase of GSH content per oocyte compared with the CPF group (Figures 5A,C, *p* < 0.05). Moreover, the expression levels of Nrf2, Sod1, Gpx4, Cat, and Gstm2 in the CPF group and only Nrf2 transcription in the CPT group were significantly downregulated compared with the CON group, and the CPT group displayed the significantly higher transcription levels of Nrf2, Gpx4, Cat, and Gstm2 than those in the CPF group. These results suggested that CPF enhanced ROS production of and decreased GSH content through downregulating the expression of redox genes in oocytes, while PTS effectively ameliorated these damages in CPF-exposed oocytes.

### The Development Competence, Quality and Apoptosis of Early Embryos Derived From Oocytes Treated With CPF Or/And PTS

**FIGURE 6:**
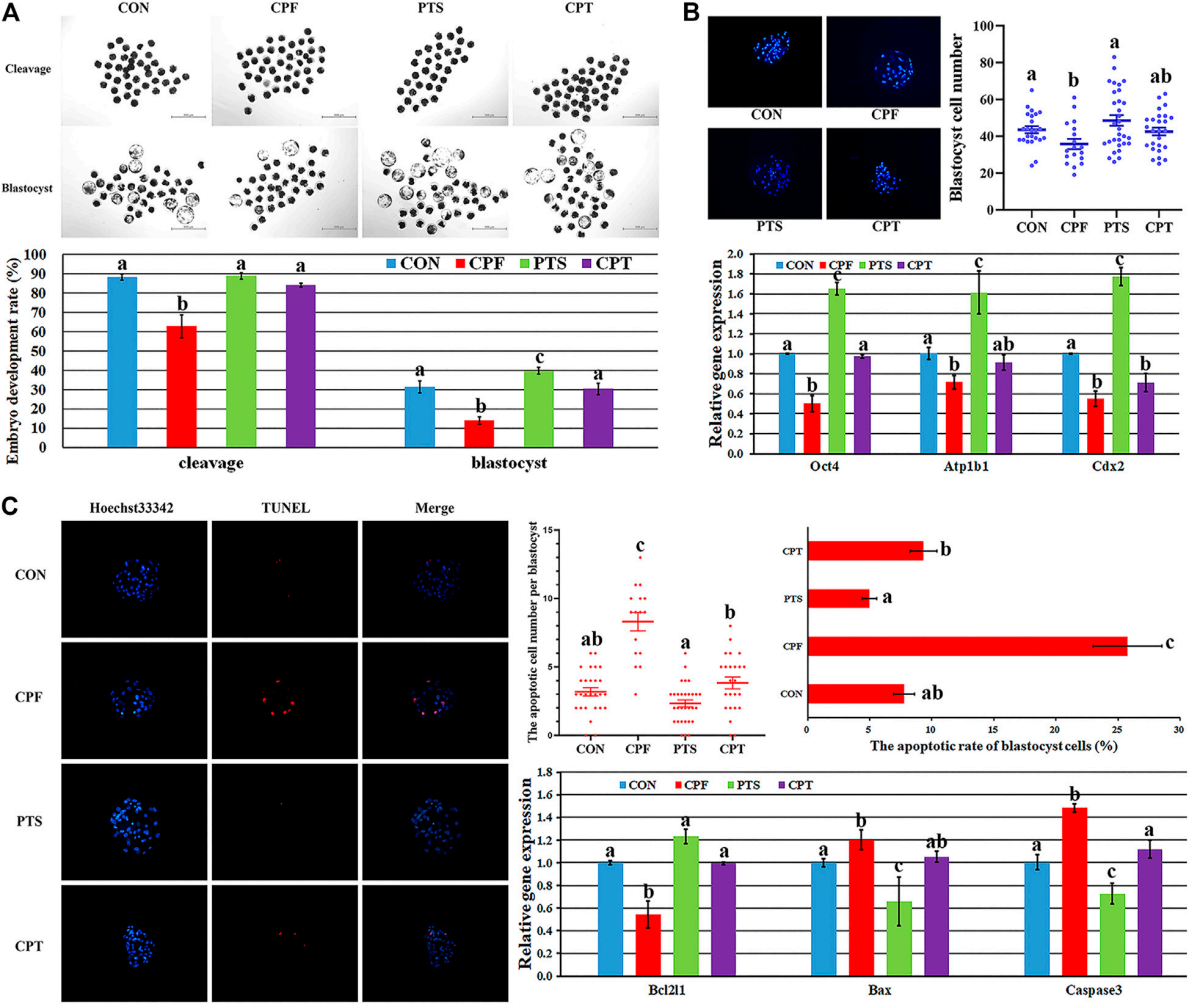
The development competence, quality and apoptosis of early embryos derived from oocytes treated with CPF or/and PTS. **(A)**, the morphologies and rates of cleavage and blastocyst. **(B)**, the morphology and number of blastocyst cells and relative expression of blastocyst quality related genes. **(C)**, the morphology, number and rate of blastocyst cell apoptosis and relative expression of apoptosis related genes. ^a-c^Values for a certain group with different superscripts differ significantly (*p* < 0.05).

The quality of oocyte cytoplasmic maturation determines the subsequent development of embryos. Here, the development competence, quality and apoptosis of parthenogenetic embryos were applied to further examine the cytoplasmic maturation of oocytes treated with CPF or/and PTS. As shown in Figures 6A,B and Supplementary Table S7, compared with the CON group, the CPF group showed the significantly decreased cleavage and blastocyst rates and blastocyst cell number (*p* < 0.05), while the CPT group displayed no significant differences of cleavage and blastocyst rates and blastocyst cell number, and the significantly upregulated percentages of cleavage and blastocyst rates were observed in the CPT group compared with the CPF group (*p* < 0.05). In addition, the expression of blastocyst quality related genes showed that the transcription levels of inner cell mass gene Oct4, blastocyst cavity gene Atp1b1 and trophectoderm gene Cdx2 were significantly downregulated in the CPF group, but only Cdx2 transcription was significantly decreased in the CPT group compared with the CON group, and the CPT group displayed the significantly higher expression level of Oct4 than the CPF group (*p* < 0.05). Moreover, compared with the CON group, the CPF group showed the significantly increased number and rate of apoptotic blastocyst cells, downregulated expression level of antiapoptotic factor Bcl2l1, and upregulated transcription levels of proapoptotic gene Bax and Caspase3 (*p* < 0.05), while the CPT group displayed the similar patterns of apoptotic blastocyst cells and gene expression. Compared with the CPF group, the significantly decreased number and rate of apoptotic blastocyst cells, upregulated Bcl2l1 expression, and downregulated Caspase3 transcription were observed in the CPT group. Taken together, oocyte treatment with CPF further reduced the subsequent development competence and quality of embryos, and caused embryo apoptosis, while PTS alleviated these defects.

## Discussion

**FIGURE 7:**
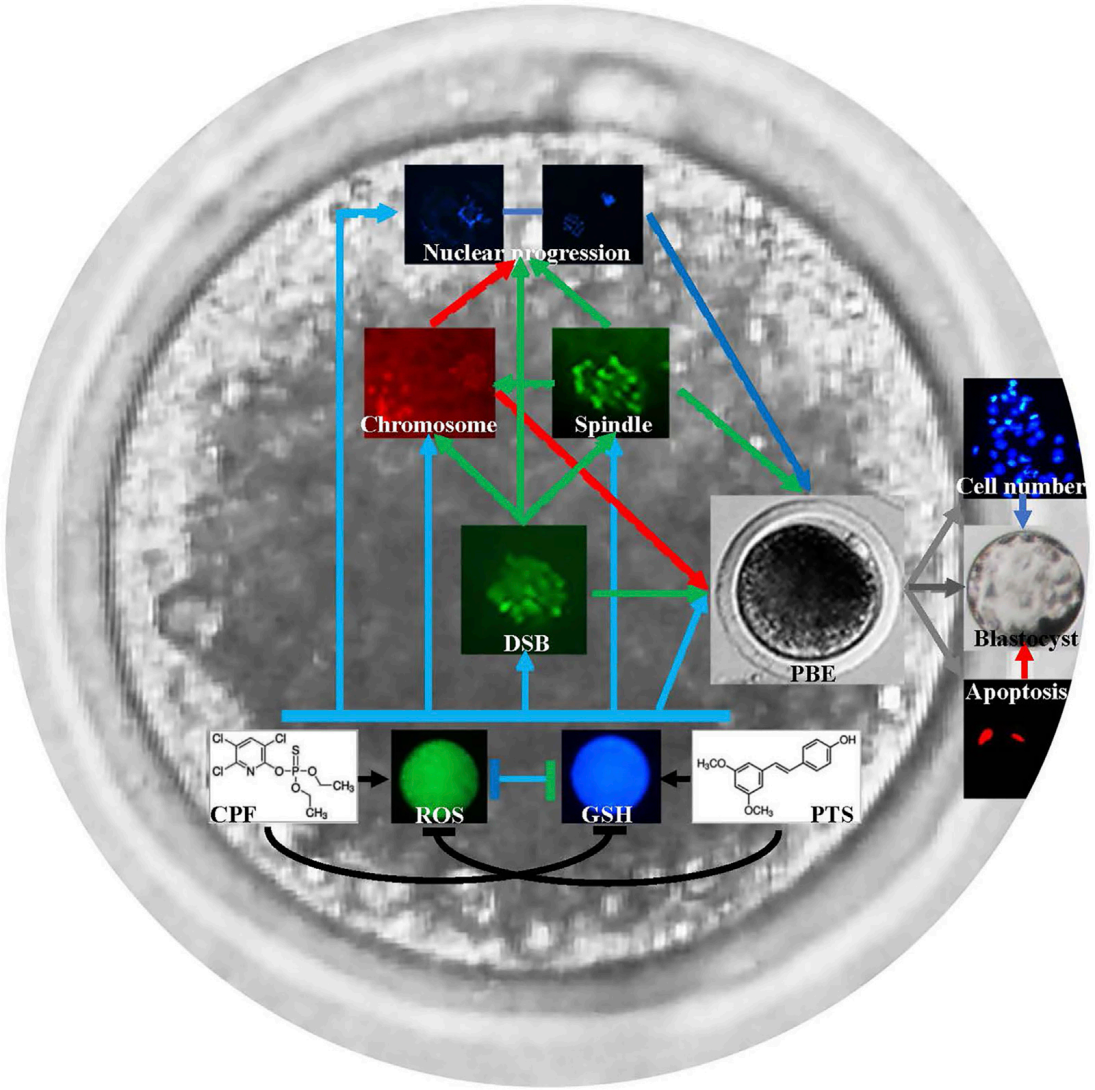
Schematic representation of the protective role of PTS against CPF to ameliorate oocyte maturation. CPF breaks the redox balance to produce ROS and reduce GSH, causes DNA damage, disrupts spindle assembly and chromosome alignment, and further destroys oocyte nuclear maturation progression and oocyte maturation. PTS rescues CPF-mediated defects and ameliorates oocyte maturation.

Oocyte maturation is an essential event in mammalian reproduction, and recently, to evaluate the potential effect of environmental compounds on oocyte maturation has been gaining attention for human and animal reproductive health (Chen et al., 2020; Jiang et al., 2021; Mourikes and Flaws, 2021). Presently, how CPF affects oocyte maturation and whether PTS can protect oocytes against CPF remain unclear. In this study, CPF was shown to exert the detrimental effect on porcine oocyte maturation, and the potential mechanism could be that CPF broke the redox balance to produce ROS and reduce GSH, caused DNA damage, disrupted spindle assembly and chromosome alignment, and destroyed oocyte nuclear maturation progression. And, PTS was also proven to effectively protect oocyte maturation against CPF-mediated defects (Figure 7).

Nowadays, the usage of OP insecticides remains worldwide and leads to the serious environmental problem (Fucic et al., 2021). Previous studies have shown that OP insecticides can make the loss of cell function and cause the defects to cells, and CPF could exert the detrimental effects to the reproductive system and the toxicology to oocytes (Nishi and Hundal, 2013; Li et al., 2015; Jiang et al., 2021). Then, to understand the adverse effects of CPF on oocytes could be particularly necessary. As PBE is a critical meiotic event to evaluate oocyte nuclear maturation, it was examined after porcine oocytes was treated with CPF. The result showed that CPF significantly blocked PBE and caused oocyte death, in consistent with the reports of harmful effect of exogenous hazardous compounds on oocyte maturation (Jiao et al., 2020; Shi et al., 2021). In addition, CPF exerted the detrimental effect on oocytes in a dose dependent manner, suggesting that there is a cumulatively toxic effect of CPF to impair oocyte maturation. Moreover, recent studies have shown that CPF causes damage to oocytes (Nandi et al., 2011; Jiang et al., 2021). Thus, CPF is detrimental to oocyte maturation.

During oocyte maturation, GV, GVBD, MI, AI/TI, and MII successively occurs. As oocyte nuclear maturation progression determines PBE, it was postulated that CPF could inhibit oocyte nuclear maturation progression, and this hypothesis was supported by the results that oocyte nuclear maturation progression was inhibited in the CPF group, with the obviously downregulated percentages of oocytes at the time points of MI, AI/TI and MII events and arrested proportions of oocytes at GVBD and MI when AI/TI and MII events occurred. The reason may be due to the interference of CPF on the expression of meiotic cell cycle factors, as meiotic progression is mainly regulated by MPF and MAPK (Cao et al., 2020). The obviously downregulated expression of Cdk1, Ccnb1, and MAPK3 confirmed this point. As for the detail interaction between CPF and meiotic cell cycle factors, it needs to be investigated. Anyhow, these results demonstrate that CPF delays or inhibits oocyte nuclear maturation progression to further reduce oocyte maturation.

PBE is also closely associated with spindle assembly and chromosome alignment (Zhang S.-X. et al., 2020). Then, the states of spindle assembly and chromosome alignment were examined to evaluate the adverse effect of CPF on oocytes. Notably, CPF was shown to significantly cause the abnormalities of spindle assembly and chromosome alignment, further supporting that the aberrant spindle assembly and chromosome misalignment reduced PBE. Meanwhile, the assembly and separation of spindle and chromosome are mainly regulated by SAC related genes (Marston and Wassmann, 2017). The obvious decrease expression of Bub1, Mad1, and Mad2 in the CPF group further suggested that CPF destroyed the normal assembly and separation of spindle and chromosome, and then delayed or inhibited the meiotic progression through MI and the release of PBE. Recent studies have also displayed that CPF exerts the detrimental effect on spindle and chromosome in sperms and oocytes (Pallotta et al., 2019; Jiang et al., 2021). Accordingly, CPF induces aberrant spindle assembly and chromosome alignment, and further impairs oocyte nuclear maturation progression and the release of PBE.

DNA damage has been proven to give rise to spindle defect, and further inhibit cell cycle progression and oocyte maturation (Wang et al., 2016). To further explore the potential mechanism of the toxicity of CPF during oocyte maturation, γ-H2AX in response to DNA DSB was used as a marker to examine DNA damage. In the CPF group, the γ-H2AX level was obviously upregulated, proving that CPF causes DNA damage to oocytes. CPF-induced DNA damage further causes oocyte spindle defect, arrests oocytes at GVBD and MI, and inhibits oocyte nuclear maturation progression. Previous studies have also shown that DNA damage disrupts genomic stability including chromosome alignment and affect oocyte maturation (Wang et al., 2016; He et al., 2018). This study further evidences that oocyte maturation is severely reduced when genome stability is impaired by DNA damage. Furthermore, the occurrence of DNA DSB triggers the process of DNA repair. It has been proven that Atm, the master regulator of DNA damage checkpoint, accumulates at DSB site, which further recruits DNA repair-related genes including Rad51 and 53bp1 to repair DSB by the homologous recombination (HR) and nonhomologous end joining (NHEJ) pathways, respectively (Wang et al., 2015). As DNA repair-related genes can repair DNA damage, it is remarkable that the significantly increased expression of Atm, Rad51 and 53bp1 in the CPF group further indicates that DNA damage is upregulated in the CPF group. It is also evidenced that CPF triggers DNA damage in cells (Li et al., 2015). Thus, CPF could be an inducer of oocyte DNA damage and reduce oocyte maturation.

ROS, as the mediator of oxidative stress, has been shown to cause DNA damage to cells, while GSH, a common free thiol intracellular compound, functions as an antioxidant to scavenge free radicals and defend cells against the damage of oxidative stress (Zhou C. et al., 2019). ROS and GSH are also known as the key markers to regulate the intracellular redox balance and evaluate oocyte cytoplasmic maturation. Here, ROS and GSH were applied to investigate the effect of CPF on oocyte maturation, and CPF was shown to significantly increase ROS and decrease GSH, suggesting that CPF impairs oocyte cytoplasmic maturation by destroying the balance between ROS and GSH, similar with the previous studies in cells (Chiapella et al., 2013; Tadee et al., 2020). The antioxidant molecules have been shown to eliminate free radicals and protect cells against oxidative damage. Nrf2 is a key factor regulating oxidative stress, and its downstream antioxidant genes including Sod1, Gpx4 and Cat and glutathione S-transferase gene Gstm2 can maintain the redox balance (Ullah et al., 2019). The reduced expression of Nrf2, Sod1, Gpx4, Cat, and Gstm2 genes in the CPF group further confirmed that CPF destroyed the redox system of oocyte cytoplasm. Thus, CPF impairs oocyte cytoplasmic maturation by breaking the redox system. Given that the increased ROS level induced by exogenous hazardous compounds has been previously shown to cause the decreased GSH content, DNA damage, spindle abnormality and chromosome misalignment (Pang et al., 2019; Jeong et al., 2020; Shi et al., 2021), it suggests that CPF induces oxidative stress by increasing ROS, which further causes the reduction of GSH content, the upregulation of DNA damage and the abnormalities of spindle assembly and chromosome alignment, then inhibits or delays oocyte maturation progression, and finally impairs oocyte maturation.

The development competence and quality of early embryos can further reflect the quality of oocyte cytoplasmic maturation. Here, the significantly downregulated cleavage and blastocyst rates in the CPF group suggested that CPF destroyed oocyte cytoplasmic maturation, and the obviously decreased blastocyst cell number also indicated that oocyte cytoplasmic maturation was impaired after CPF treatment. Moreover, the reduced blastocyst quality reflected by the significantly decreased expression of inner cell mass gene Oct4, blastocyst cavity gene Atp1b1 and trophectoderm gene Cdx2 further demonstrated that CPF was detrimental to oocyte cytoplasmic maturation. The reduced development competence and quality of early embryos could be attributed to apoptosis caused by CPF, as embryos do not repair the damage but process to apoptosis or arrest, and apoptosis negatively affect embryo quality (Wang et al., 2021). Notably, CPF was shown to significantly trigger embryo apoptosis, further supporting that the reduced blastocyst quality was induced by the increased apoptosis in the CPF group. The disrupted expression patterns of anti-apoptosis gene Bcl2l1 and apoptosis genes including Bax and Caspase3 also confirmed that CPF destroyed the anti-apoptosis signaling pathway to reduce blastocyst cell number. Thus, CPF impaired oocyte cytoplasmic maturation reflected by the decreased embryo development competence and quality, and increased embryo apoptosis.

Natural antioxidants have been shown to take the protective role during oocyte maturation, and previous studies have also highlighted that PTS has the powerful antioxidant effect to protect cells against the damage caused by hazardous compounds (Acharya and Ghaskadbi, 2013; Ullah et al., 2019; Tang et al., 2021). Here, when PTS was applied to treat CPF-exposed oocytes, GSH level was significantly increased, ROS and DNA damage were remarkedly reduced, spindle assembly and chromosome alignment were obviously ameliorated, oocyte nuclear maturation progression was also largely rescued, and, importantly, oocyte maturation and subsequent embryo development competence and quality were improved, further supporting that PTS possesses the ability to protect oocytes against damage caused by CPF. Previous studies have also displayed that PTS can increase GSH content, decrease ROS level and reduces DNA damage in cells (Acharya and Ghaskadbi, 2013; Ullah et al., 2019). Thus, PTS can protect oocyte maturation against CPF-induced damage. As how PTS antagonizes CPF to rescue the defects during oocyte maturation, especially the detail molecular mechanism underlying the redox system, spindle assembly, chromosome alignment and DNA damage, the possible pathway might be that PTS could ameliorate gene expression pattern related to oocyte maturation. Of course, the regulatory network of gene expression is complex, and further studies are needed. All in all, PTS effectively alleviated the detrimental effect of CPF on oocyte maturation.

In conclusion, this study displays that CPF reduces oocyte maturation in pigs, and the underlying mechanism is that CPF breaks the redox balance to produce ROS and reduce GSH, disrupts spindle assembly and chromosome alignment, causes DNA damage, and destroys oocyte nuclear maturation progression. Notably, PTS is also shown to effectively rescue CPF-mediated defect during oocyte maturation.

## Data Availability

The original contributions presented in the study are included in the article/Supplementary Material, further inquiries can be directed to the corresponding author.
