# Supplementary material for: Pterostilbene Alleviates Chlorpyrifos-Induced Damage During Porcine Oocyte Maturation
Source: Front Cell Dev Biol. 2021 Dec 21;9:803181. doi: 10.3389/fcell.2021.803181 (PMC8724426; doi:10.3389/fcell.2021.803181)
Supplement: Supplementary file 1 [file Table1.DOCX]

**Supplemental material**

**Tables**

**Table S1.** Detail of primers for quantitative real-time PCR

| Gene | Primer sequence (5'-3') | Length (bp) | | Accession number |
| --- | --- | --- | --- | --- |
| Cdk1 | F: GGCACTCCCAATAATGAAGTGTG | 103 | | NM_001159304 |
|  | R: TTTTGACGTGGGATGCTAGGC |  |  |  |
| Ccnb1 | F: CTTCGGAGAGCATCCAAGATTG | 111 | | NM_001170768 |
|  | R: AGAAGGAGGAAAGTGCACCATATC |  |  |  |
| Mapk3 | F: CAACGACCACATCTGCTACTTCC | 126 | | XM_021088019 |
|  | R: TTAAGGTCGCAGGTGGTGTTG |  |  |  |
| Bub1 | F: TTCTAAGCCAGTGAGTTCCTATCC | 183 | | XM_021087046 |
|  | R: TTGTTTTTGTTATCGTTCACATCC |  |  |  |
| Mad1 | F: TCCTTGAACAGCTTCATCTCTCAG | 201 | | XM_021085975 |
|  | R: TCGAGCCCTCTTGTGACTCAG |  |  |  |
| Mad2 | F: AGTTCTTCTCGTTTGGCATCAAC | 153 | | XM_003129223 |
|  | R: TTAGTTGTTCCACCACATTATTTAGG |  |  |  |
| Atm | F: AAAAACAGGCTTTATTCGCCCT | 162 | | NM_001123080 |
|  | R: GTAGCCATTCCAAAACCAGATAATC |  |  |  |
| Rad51 | F: CACAGCCTATTTCACGATTAGAGCA | 146 | | NM_001123181 |
|  | R: TTTGGCTTCACTAATCCCCTTAATA |  |  |  |
| 53bp1 | F: AAATGCTCAGATTCCCAGTCG | 131 | | XM_021096915 |
|  | R: TCTTTTTCTGCAGTTACTGATTTCA |  |  |  |
| Nrf2 | F: CTGCCGACTATTCCCAGGTAG  R: GCCGAAGAAACCTCATTGTCATC | 118 | | MH_101365 |
| Sod1 | F: TTGGAGACCTGGGCAATGTGAC | 184 | | NM_001190422 |
|  | R: CTTCCAGCATTTCCCGTCTTTGTA |  |  |  |
| Gpx4 | F: ATGCACGAATTCTCAGCCAAG | 122 | NM_214407 | |
|  | R: GTGTAGTTTACCTCCGTCTTGCC |  |  |  |
| Cat | F: AGGTGGGGCTCCAAATTACTAC | 136 | NM_214301 | |
|  | R: CACCTGGGTGACATTATCTTCG |  |  |  |
| Gstm2 | F: CTTTGAGAAACTGAAGCCTGGGTAC | 159 | DQ_988117 | |
|  | R: GGATCGAATATGCGGTACAGGTC |  |  |  |
| Oct4 | F: GAAGGTGTTCAGCCAAACGAC | 185 | NM_001113060 | |
|  | R: CGATACTTGTCCGCTTTC |  |  |  |
| Atp1b1 | F: CCACCAGGATTAACACAGATTCC | 185 | NM_001001542 | |
|  | R: TCTTTGAGTTCGCTGGGCAC |  |  |  |
| Cdx2 | F: GACAAGGACGTGAGCATGTATCC | 220 | XM_003130908 | |
|  | R: CGTAGCCGTTCCAGTCCTCG |  |  |  |
| Bcl2l1 | F: CTGGTGGTTGACTTTCTCTCCTAC | 119 | NM_214285 | |
|  | R: GTTTCCGCTTCTGATTCAGTCC |  |  |  |
| Bax | F: CAGTAACATGGAGCTGCAGAGG | 159 | XM_003127290 | |
|  | R: GCCTTGAGCACCAGTTTACTGG |  |  |  |
| Caspase3 | F: CAGTTGAGGCAGACTTCTTGTATG | 149 | NM_214131 | |
|  | R: AGTAAGAATGTGCATAAGCTCAAGC |  |  |  |
| 18s | F: AATCTCGGGTGGCTGAACGC | 143 | NR_002170 | |
|  | R: CCGTTCTTAGTTGGTGGAGCGAT |  |  |  |

**Table S2.** Maturation of oocytes treated with various concentration CPF

| CPF (μM) | No. oocytes (Rep) | No. oocytes matured (% ± SEM) | No. oocytes dead (% ± SEM) |
| --- | --- | --- | --- |
| 0.00 | 294 (5) | 213 (72.06 ± 2.91)^a^ | 31 (10.77 ± 1.02)^a^ |
| 0.05 | 304 (5) | 207 (67.66 ± 1.66)^a^ | 38 (12.65 ± 1.35)^a^ |
| 0.10 | 290 (5) | 192 (65.90 ± 2.82)^ab^ | 43 (15.00 ± 1.52)^ab^ |
| 0.25 | 299 (5) | 177 (58.91 ± 2.16)^bc^ | 55 (18.62 ± 1.52)^b^ |
| 0.50 | 274 (5) | 145 (52.92 ± 2.71)^cd^ | 74 (27.03 ± 3.19)^c^ |
| 1.00 | 285 (5) | 145 (50.78 ± 2.27)^cd^ | 88 (31.00 ± 2.08)^c^ |

^a-d^Values in the same column with different superscripts differ significantly (P<0.05).

**Table S3.** Maturation of oocytes treated with various concentration PTS

| PTS (μM) | No. oocytes (Rep) | No. oocytes matured (% ± SEM) | No. oocytes dead (% ± SEM) |
| --- | --- | --- | --- |
| 0.00 | 283 (5) | 204 (72.08 ± 1.37)^a^ | 30 (10.61 ± 0.22) |
| 0.10 | 269 (5) | 201 (74.49 ± 1.83)^ab^ | 27 (10.09 ± 0.81) |
| 0.25 | 285 (5) | 215 (75.13 ± 2.80)^ab^ | 30 (10.68 ± 1.49) |
| 0.50 | 285 (5) | 222 (78.17 ± 1.83)^b^ | 29 (10.18 ± 1.92) |
| 0.75 | 278 (5) | 216 (77.92 ± 1.85)^b^ | 32 (11.45 ± 1.36) |
| 1.00 | 302 (5) | 232 (76.88 ± 1.73)^ab^ | 36 (11.93 ± 0.92) |

^a-b^Values in the same column with different superscripts differ significantly (P<0.05).

**Table S4.** Maturation of oocytes treated with CPF or/and PTS

| Group | No. oocytes (Rep) | No. oocytes matured (% ± SEM) | No. oocytes immatured (% ± SEM) | No. oocytes dead (% ± SEM) |
| --- | --- | --- | --- | --- |
| CON | 336 (5) | 245 (72.73 ± 2.09)^a^ | 55 (16.46 ± 1.00)^a^ | 36 (10.81 ± 2.10)^ac^ |
| CPF | 290 (5) | 166 (57.10 ± 2.22)^b^ | 64 (22.15 ± 2.04)^b^ | 60 (20.75 ± 0.37)^b^ |
| PTS | 259 (5) | 203 (78.52 ± 1.56)^c^ | 30 (11.52 ± 1.10)^c^ | 26 (9.95 ± 1.03)^a^ |
| CPT | 296 (5) | 202 (68.19 ± 1.65)^a^ | 51 (17.21 ± 1.49)^a^ | 43 (14.60 ± 1.38)^c^ |

^a-c^Values in the same column with different superscripts differ significantly (P<0.05).

**Table S5.** Oocyte nuclear state during the maturation progress

| Time (h) | No. oocytes (Rep) | No. GV oocytes (%±SEM) | No. GVBD oocytes (%±SEM) | No. MI oocytes (%±SEM) | No. AI/TI oocytes (%±SEM) | No. MII oocytes (%±SEM) | No. AII/TII oocytes (%±SEM) |
| --- | --- | --- | --- | --- | --- | --- | --- |
| 0 | 155 (3) | 155 (100.00±0.00)^a^ | 0 (0.00±0.00)^a^ | 0 (0.00±0.00)^a^ | 0 (0.00±0.00)^a^ | 0 (0.00±0.00)^a^ | 0 (0.00±0.00)^a^ |
| 12 | 133 (3) | 102 (76.63±1.24)^b^ | 31 (23.37±1.24)^b^ | 0 (0.00±0.00)^a^ | 0 (0.00±0.00)^a^ | 0 (0.00±0.00)^a^ | 0 (0.00±0.00)^a^ |
| 18 | 138 (3) | 39 (28.27±0.67)^c^ | 92 (66.70±2.00)^cd^ | 7 (5.03±1.40)^bc^ | 0 (0.00±0.00)^a^ | 0 (0.00±0.00)^a^ | 0 (0.00±0.00)^a^ |
| 19 | 136 (3) | 24 (17.72±1.64)^d^ | 102 (75.04±1.44)^e^ | 10 (7.24±1.69)^bc^ | 0 (0.00±0.00)^a^ | 0 (0.00±0.00)^a^ | 0 (0.00±0.00)^a^ |
| 20 | 136 (3) | 21 (15.41±1.03)^e^ | 93 (68.47±1.99)^c^ | 22 (16.12±1.09)^d^ | 0 (0.00±0.00)^a^ | 0 (0.00±0.00)^a^ | 0 (0.00±0.00)^a^ |
| 21 | 124 (3) | 10 (8.06±0.76)^f^ | 78 (62.79±1.92)^d^ | 34 (27.57±2.92)^e^ | 2 (1.57±0.78)^ab^ | 0 (0.00±0.00)^a^ | 0 (0.00±0.00)^a^ |
| 23 | 130 (3) | 4 (3.05±0.70)^g^ | 51 (39.32±2.11)^f^ | 70 (53.84±1.02)^f^ | 5 (3.79±1.44)^bc^ | 0 (0.00±0.00)^a^ | 0 (0.00±0.00)^a^ |
| 24 | 124 (3) | 1 (0.85±0.85)^h^ | 31 (24.97±1.80)^b^ | 84 (67.70±1.03)^gh^ | 8 (6.48±0.88)^c^ | 0 (0.00±0.00)^a^ | 0 (0.00±0.00)^a^ |
| 25 | 123 (3) | 0 (0.00±0.00)^h^ | 22 (17.81±1.72)^g^ | 87 (70.81±2.22)^g^ | 13 (10.54±0.54)^d^ | 1 (0.83±0.83)^a^ | 0 (0.00±0.00)^a^ |
| 26 | 126 (3) | 1 (0.78±0.78)^h^ | 17 (13.37±1.66)^h^ | 80 (63.69±2.78)^h^ | 26 (20.55±1.53)^e^ | 2 (1.61±0.81)^a^ | 0 (0.00±0.00)^a^ |
| 29 | 127 (3) | 0 (0.00±0.00)^h^ | 6 (4.78±1.49)^ij^ | 36 (28.32±0.42)^e^ | 75 (59.05±0.95)^f^ | 10 (7.86±2.08)^b^ | 0 (0.00±0.00)^a^ |
| 30 | 127 (3) | 0 (0.00±0.00)^h^ | 7 (5.40±1.88)^i^ | 23 (18.19±1.32)^d^ | 85 (66.87±1.01)^g^ | 12 (9.54±1.60)^b^ | 0 (0.00±0.00)^a^ |
| 31 | 125 (3) | 0 (0.00±0.00)^h^ | 1 (0.74±0.74)^aj^ | 11 (8.80±0.72)^c^ | 87 (69.54±1.07)^g^ | 26 (20.93±1.57)^c^ | 0 (0.00±0.00)^a^ |
| 32 | 123 (3) | 0 (0.00±0.00)^h^ | 2 (1.67±0.83)^ai^ | 5 (4.07±0.83)^ab^ | 69 (56.03±2.68)^f^ | 47 (38.23±2.85)^d^ | 0 (0.00±0.00)^a^ |
| 36 | 133 (3) | 0 (0.00±0.00)^h^ | 0 (0.00±0.00)^a^ | 7 (5.27±0.77)^bc^ | 24 (18.05±1.32)^eh^ | 100 (75.17±2.36)^e^ | 2 (1.52±0.76)^b^ |
| 42 | 128 (3) | 0 (0.00±0.00)^h^ | 0 (0.00±0.00)^a^ | 6 (4.67±1.33)^bc^ | 19 (14.82±1.98)^h^ | 98 (76.60±2.56)^e^ | 5 (3.91±0.79)^c^ |

^a-j^Values in the same column with different superscripts differ significantly (P<0.05).

**Table S6.** Effect of CPF and PTS on oocyte nuclear state during the maturation progress

| Time (h) | Group | No. oocytes (Rep) | No. GV oocytes (%±SEM) | No. GVBD oocytes (%±SEM) | No. MI oocytes (%±SEM) | No. AI/TI oocytes (%±SEM) | No. MII oocytes (%±SEM) | No. AII/TII oocytes (%±SEM) |
| --- | --- | --- | --- | --- | --- | --- | --- | --- |
| 19 | CON | 138 (3) | 25 (18.32±1.41)^a^ | 104 (75.44±1.27) | 9 (6.25±1.85) | 0 (0.00±0.00) | 0 (0.00±0.00) | 0 (0.00±0.00) |
|  | CPF | 125 (3) | 16 (12.98±1.30)^b^ | 101 (80.65±1.53) | 8 (6.37±0.51) | 0 (0.00±0.00) | 0 (0.00±0.00) | 0 (0.00±0.00) |
|  | PTS | 138 (3) | 28 (20.22±1.41)^a^ | 103 (74.79±3.18) | 7 (4.98±1.78) | 0 (0.00±0.00) | 0 (0.00±0.00) | 0 (0.00±0.00) |
|  | CPT | 133 (3) | 25 (18.74±1.05)^a^ | 100 (75.29±1.98) | 8 (5.97±1.37) | 0 (0.00±0.00) | 0 (0.00±0.00) | 0 (0.00±0.00) |
| 25 | CON | 141 (3) | 2 (1.44±0.73)^ab^ | 22 (15.59±1.62)^a^ | 102 (72.39±2.63)^a^ | 15 (10.57±0.78)^a^ | 0 (0.00±0.00) | 0 (0.00±0.00) |
|  | CPF | 121 (3) | 4 (3.30±0.79)^a^ | 33 (27.45±3.59)^b^ | 78 (64.35±1.93)^b^ | 6 (4.90±1.35)^b^ | 0 (0.00±0.00) | 0 (0.00±0.00) |
|  | PTS | 139 (3) | 1 (0.67±0.67)^b^ | 19 (13.72±0.94)^a^ | 106 (76.22±1.21)^a^ | 13 (9.39±0.83)^a^ | 0 (0.00±0.00) | 0 (0.00±0.00) |
|  | CPT | 135 (3) | 3 (2.22±0.05)^ab^ | 26 (19.19±1.59)^a^ | 95 (70.46±2.73)^ab^ | 11 (8.12±1.38)^ab^ | 0 (0.00±0.00) | 0 (0.00±0.00) |
| 31 | CON | 142 (3) | 0 (0.00±0.00) | 1 (0.67±0.67)^ab^ | 11 (7.80±1.54)^ab^ | 101 (71.04±1.48)^ab^ | 29 (20.49±1.77)^a^ | 0 (0.00±0.00) |
|  | CPF | 119 (3) | 1 (0.81±0.81) | 13 (10.99±1.85)^c^ | 33 (27.67±2.05)^c^ | 60 (50.43±1.09)^c^ | 12 (10.09±0.23)^b^ | 0 (0.00±0.00) |
|  | PTS | 138 (3) | 0 (0.00±0.00) | 0 (0.00±0.00)^a^ | 8 (5.72±1.22)^a^ | 104 (75.29±1.23)^a^ | 26 (18.99±2.41)^a^ | 0 (0.00±0.00) |
|  | CPT | 131 (3) | 0 (0.00±0.00) | 5 (3.82±0.77)^b^ | 18 (13.76±2.31)^b^ | 86 (65.74±3.22)^b^ | 22 (16.69±1.32)^a^ | 0 (0.00±0.00) |
| 42 | CON | 131 (3) | 0 (0.00±0.00) | 0 (0.00±0.00)^a^ | 8 (5.97±1.86)^ab^ | 19 (14.64±1.97) | 100 (76.26±2.14)^ab^ | 4 (3.13±0.93)^ab^ |
|  | CPF | 115 (3) | 0 (0.00±0.00) | 6 (5.27±0.36)^b^ | 21 (18.02±1.81)^c^ | 12 (10.63±1.87) | 69 (59.84±1.50)^c^ | 7 (6.25±1.30)^a^ |
|  | PTS | 130 (3) | 0 (0.00±0.00) | 0 (0.00±0.00)^a^ | 3 (2.29±1.29)^a^ | 18 (13.74±1.99) | 105 (80.90±2.29)^a^ | 4 (3.07±0.74)^b^ |
|  | CPT | 123 (3) | 0 (0.00±0.00) | 1 (0.95±0.95)^a^ | 13 (10.12±1.67)^b^ | 16 (12.64±1.37) | 88 (72.20±2.45)^b^ | 5 (4.09±0.79)^ab^ |

^a-d^Values at the same time in the same column with different superscripts differ significantly (P<0.05).

**Table S7.** The development of embryos derived from oocytes treated with CPF or/and PTS

| Group | No. embryos (Rep) | No. embryos cleaved (% ± SEM) | No. blastocysts (% ± SEM) | Blastocyst cell number (mean ± SEM)* |
| --- | --- | --- | --- | --- |
| CON | 109 (3) | 96 (88.27 ± 1.69)^a^ | 34 (31.51 ± 3.12)^a^ | 44 ± 2 (n=24)^a^ |
| CPF | 107 (3) | 67 (62.87 ± 5.87)^b^ | 15 (14.09 ± 1.91)^b^ | 36 ± 3 (n=18)^b^ |
| PTS | 118 (3) | 105 (89.00 ± 1.66)^a^ | 47 (39.85 ± 1.89)^c^ | 49 ± 3 (n=31)^a^ |
| CPT | 121 (3) | 102 (84.26 ± 1.12)^a^ | 37 (30.45 ± 2.92)^a^ | 43 ± 2 (n=26)^ab^ |

^a-c^Values in the same column with different superscripts differ significantly (P<0.05).
